# Mirrors and toothaches: commonplace manipulations of non-auditory feedback availability change perceived speech intelligibility

**DOI:** 10.3389/fnhum.2024.1462922

**Published:** 2024-11-27

**Authors:** Elizabeth D. Casserly, Francesca R. Marino

**Affiliations:** ^1^Department of Psychology and Neuroscience Program, Trinity College, Hartford, CT, United States; ^2^Department of Anatomy and Neurobiology, Boston University School of Medicine, Boston, MA, United States

**Keywords:** multisensory integration, speech motor control, somatosensory feedback, visual feedback, speech intelligibility

## Abstract

This paper investigates the impact of two non-technical speech feedback perturbations outside the auditory modality: topical application of commercially-available benzocaine to reduce somatosensory feedback from speakers’ lips and tongue tip, and the presence of a mirror to provide fully-detailed visual self-feedback. In experiment 1, speakers were recorded under normal quiet conditions (i.e., baseline), then again with benzocaine application plus auditory degradation, and finally with the addition of mirror feedback. Speech produced under normal and both feedback-altered conditions was assessed via naïve listeners’ intelligibility discrimination judgments. Listeners judged speech produced under bisensory degradation to be less intelligible than speech from the un-degraded baseline, and with a greater degree of difference than previously observed with auditory-only degradation. The introduction of mirror feedback, however, did not result in relative improvements in intelligibility. Experiment 2, therefore, assessed the effect of a mirror on speech intelligibility in isolation with no other sensory feedback manipulations. Speech was recorded at baseline and then again in front of a mirror, and relative intelligibility was discriminated by naïve listeners. Speech produced with mirror feedback was judged as less intelligible than baseline tokens, indicating a negative impact of visual self-feedback in the absence of other sensory manipulations. The results of both experiments demonstrate that relatively accessible manipulations of non-auditory sensory feedback can produce speech-relevant effects, and that those effects are perceptible to naïve listeners.

## 1 Introduction

Perturbation of sensory feedback during real-time speech production is a powerful methodological tool. It allows us to probe the temporal boundaries of feedback integration (Behroozmand et al., 2016), examine individual differences in and control of feedback sensitivity (Munhall et al., 2009), and build models of real-time speech motor control that are grounded in both behavior and its neurological correlates (Guenther and Vladusich, 2012; Hickok, 2013; Houde and Nagarajan, 2011; Parrell and Houde, 2019). As specified in such models, speech articulation likely involves a combination of feedforward control, where pre-determined articulation plans proceed without moment-to-moment correction, and longer timescales of adjustment based on sensory feedback; the relatively intelligible speech produced by adults experiencing acute hearing loss (Gould et al., 2001) and the speed with which fluent articulatory movements are executed, demonstrates the robustness of feedforward systems in real-time speech motor control (Guenther and Vladusich, 2012). Despite this robustness, however, speakers reliably alter their articulation under conditions of perturbed feedback (Houde and Jordan, 1998; Lane and Tranel, 1971). Exploration of speakers’ sensitivity and responses to acoustic feedback shifts have given us valuable evidence concerning control differences across linguistic constructs (Casserly, 2011; Elman, 1981; Houde and Jordan, 1998), levels of phonological contrastivity (Jones and Munhall, 2002; Niziolek and Guenther, 2013), and production disorders in clinical populations (Demopoulos et al., 2018; Mollaei et al., 2013; Sares et al., 2018), among many other topics.

Yet speech is inherently a multisensory phenomenon, and manipulations of feedback in complementary modalities are crucial for creating and testing theories of complete integrative control of speech production.

### 1.1 Somatosensory speech feedback

Articulation produces somatosensory stimulation, even in cases where auditory feedback is unavailable or intentionally avoided (Kent, 2024). This stimulation is particularly robust to interference from environmental conditions. For example, while noise may make acoustic feedback difficult to detect or energetically mask aspects of the speech signal, those circumstances do not affect the availability or quality of somatosensory feedback. Environmental acoustics can be useful for context-specific auditory control (e.g., Hazan et al., 2012), but such interference and adaptation is much rarer for somatosensory control. As a result, it has been argued that somatosensory feedback causes an anchoring or limitation on the degree of compensation to auditory feedback perturbations (Katseff et al., 2012; Lametti et al., 2012) and may form the basis of ongoing speech intelligibility following profound hearing loss or other forms of severely disrupted hearing (Nasir and Ostry, 2008).

**FIGURE 1:**
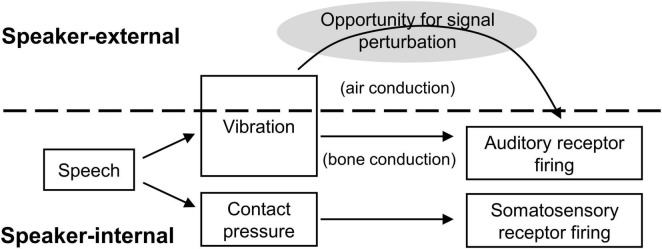
Schematic of signal transmission conditions for acoustic and somatosensory feedback. Somatosensory feedback generation and transmission occurs entirely internal to the speaker, providing no obvious opportunity for signal interception and experimental manipulation prior to sensory reception.

Feedback perturbation research in the somatosensory modality, however, has lagged behind its parallel in audition. Figure 1 illustrates the problem. With acoustic feedback, one of the primary sensory transmission pathways is external to the articulatory apparatus (*i.e.*, through vibration of the ambient medium). The stream of sensory information can therefore be disrupted externally, before reaching relevant receptors, and a perturbation can be introduced. Critically, the perturbations do not change the physical speech circumstances; it can be made to *sound* like speakers are unable to produce [s], consistently misarticulating with lower-frequency [∫], without actually impeding articulatory space. Speakers therefore have the full range of possible compensatory behaviors available, and the sensory cause of any compensation (or other behavior) is precisely known, as no other aspects of the speaking situation have changed.

Such an abstract interruption is more challenging for oral somatosensory feedback. The source of somatosensory stimulation is depression of soft tissue in the oral cavity: a deformation of the tongue, lips, palate, or gums. The change in soft tissue configuration (the stimulation source) is precisely adjacent to its receptors, so it effectively cannot be intercepted and altered like a sound wave between the oral cavity and the ears. Moreover, any attempt to physically intercede between the source and its receptors necessarily changes the action space of articulation. For example, a plastic mold of the hard palate could be introduced to eliminate or disrupt sensation from the palate during production of consonants like [k] and [t]. But as anyone who has worn an orthodontic device can attest, and as experimental studies have confirmed, the addition of even a thin palate prosthesis changes the articulatory configurations necessary for speech, particularly for sibilants like [s] and [∫] (e.g., Baum and McFarland, 1997).

Such changes to the physical articulatory space can have dramatic effects on speech production, effects which have often been studied in relation to feedback-based control. Speakers’ compensation (or lack of compensation) has been documented for devices such as palate prostheses, bite blocks, and lip tubes that modify the oral cavity or artificially hold parameters like jaw height constant (Baum and McFarland, 1997; Fowler and Turvey, 1981; Savariaux et al., 1995).

Normally, feedback from the auditory and somatosensory modalities simultaneously provide information about an alteration, its consequences for speech production, and the effectiveness of any attempted compensatory changes on the part of the speaker. The relative contributions of the two modalities in control has been controversial, however, with some results pointing to relatively weak contributions of acoustic feedback in oral configuration perturbations (Fowler and Turvey, 1981; Honda et al., 2002), while others have shown stronger influence when somatosensory effects were minimized (Jones and Munhall, 2003), and models attempt to capture the variability and interplay between the two sensory domains (e.g., Patri et al., 2019). Physical perturbations are therefore an important tool in the investigation of control and trade-offs between the acoustic and somatosensory modalities, but though they affect somatosensory feedback availability, they also impact the functional possibilities of the speech motor system more generally.

One promising method for isolated somatosensory feedback investigation has been reported (Lametti et al., 2012; Nasir and Ostry, 2008; Tremblay et al., 2003). In these studies, a robotic arm was affixed to the jaw via a custom-fitted dental device that connected to teeth on a speaker’s jaw. During articulation, a load was then applied that pulled the jaw horizontally away from the speaker’s neck by a few millimeters. Crucially, this displacement did not systematically alter the acoustic formant frequencies in the participant’s speech and was not detected by a small group of listeners in a perceptual discrimination study (Tremblay et al., 2003). It therefore produced a change that was not easily detected via acoustic feedback, unlike other articulatory configuration alterations discussed above. As a result, compensations in jaw position must have been initiated on the basis of other feedback systems. The precise source of that feedback is somewhat unclear, as it could have been either somatosensory or proprioceptive, and its locus may have been in either the jaw musculature or the oral cavity itself. Speakers typically compensated for the perturbation (Tremblay et al., 2003), and the degree of compensation was even found to stand in a complementary relationship to the degree of *acoustic* feedback perturbation speakers produced in a vowel-formant shifting context (Lametti et al., 2012).

While the horizontal jaw displacement method clearly taps into non-auditory feedback control, it does not completely solve the problem of isolated signal perturbation: the articulatory space itself was altered along with somatosensory feedback. “Pure” somatosensory feedback perturbation may be closer to the numbing performed in Larson et al. (2008). In their study of pitch feedback perturbation, participants received an aerosolized spray of either mixed anesthetic agents (benzocaine and tetracaine, 2/19 speakers) near the vocal folds or 4% lidocaine solution (17/19 speakers) directly on the vocal folds via scope application. In either case, a physician administered the anesthetic, and the targeted vocal fold application required insertion of a scope through the nasal-pharyngeal passage for the majority of speakers. Larson and colleagues observed greater *F0* compensation responses to pitch shifted acoustics under conditions of degraded somatosensory feedback, and used their results to motivate a linear integration model of feedback across the two modalities for laryngeal control (Larson et al., 2008).

Such methods interrupt the transmission of somatosensory feedback in a way that parallels the introduction of high-amplitude noise to limit acoustic feedback availability, and the anesthetic does so without altering articulatory space. There are challenges associated with these perturbation methods, however, particularly the need for specialized application techniques and personnel. Both of these challenges are greatly reduced when topical anesthetic is applied to more accessible supra-laryngeal vocal tract structures, as in De Letter et al. (2020), where a 10% lidocaine spray was applied to all of speakers’ accessible orobuccal soft tissue, including gums and soft palate, until total absence of light-touch sensation was achieved. De Letter and colleagues did not describe specialized medical monitoring or treatment of their participants under this level of topical anesthesia, although the study was conducted in a hospital setting, underwent ethics review associated with the university hospital, and its authors included a medical doctor (2020). When speakers were fully anesthetized, their articulation rate dropped and more errors were observed in a standardized nonword production task, particularly among consonant features such as place and manner of articulation (De Letter et al., 2020). Topical oral anesthetic application therefore appears to have strong potential as a method for isolated manipulation of somatosensory feedback, but such use of topical anesthetics remain relatively under-investigated, and not yet attempted outside of a medical setting or with preparation strengths available in the United States without a physician’s prescription.

### 1.2 Visual speech feedback

Self-produced speech typically does not create much in the way of immediate visual feedback. The visual field may shift in connection with speech-related head movements, and speakers can observe their own manual gestures, but the articulation process itself is not visible to a speaker under normal conditions. However, there are two reasons to believe that vision is still important to consider for feedback control. First, when visual information has been made available, speakers appear to be able to use it to aid articulation; and second, speakers are highly practiced and adept at using visual information to aid in their perception of others’ speech.

Visual self-feedback in the form of a mirror has been widely used for therapeutic interventions outside of speech, particularly in the domains of self-image rehabilitation and motor learning following stroke (Freysteinson, 2009; Gandhi et al., 2020; Thieme et al., 2019). Within the speech domain, mirrors are a traditional therapeutic tool of speech-language pathology, and still constitute an important part of how speech therapists work with patients on their articulation accuracy (American Speech-Language-Hearing Association, 2024; Ben-David and Icht, 2018; Hamerliñska and Kieczmer, 2020). More abstract visual feedback, such as ultrasound views of tongue posture, have also been influential tools for speech pathology treatment (Bernhardt et al., 2005; Bernhardt et al., 2003; Sugden et al., 2019). Outside of pathological articulation, ultrasound and visual digital displays of formants and articulator positions have also been shown to improve production accuracy in non-native adult language learners (Bliss et al., 2018; Olson and Offerman, 2021). Computerized displays of pitch, timbre, and other vocal dimensions are used in voice training for singing (Hoppe et al., 2006), and relatively simple visual feedback for vocal pitch has even appeared in popular mass media applications such as video games involving singing along to popular music (e.g., *Karaoke Revolution*, Konami Corp.). Such popular entertainment adoptions, along with the clinical and pedagogical successes reviewed above (e.g., Bernhardt et al., 2005; Hoppe et al., 2006; Olson and Offerman, 2021), suggest that speakers are quite capable of learning to use novel visual information to influence real-time speech motor control. It is important, therefore, to understand how this information is integrated, especially with respect to more typical feedback from the other senses (cf. Venezia et al., 2016).

Outside of research on speech motor control, the impact of visual information on general speech perception (i.e., other-focused perception) has been well-documented. The presence of visual speech improves recognition accuracy in adverse conditions such as environmental noise (Sumby and Pollack, 1954), disambiguates acoustically-underspecified attributes like stop place of articulation for listeners (Massaro et al., 1993), even to the point of inducing phenomena like the McGurk effect (McGurk and McDonald, 1976), and interferes with or improves recognition of individual speakers (Campanella and Belin, 2007). Integration of visual and auditory information has been argued to occur at very early stages of processing (Molholm et al., 2002; Rosenblum, 2005) and has substantial “down-stream” effects, such as facilitation of comprehension when linguistic messages are audiovisual versus purely auditory (Arnold and Hill, 2001). In short, it can be argued that perception of *other talkers’* speech is bimodal (audio-visual) at its core (e.g., Rosenblum, 2005), and that perception in either independent sensory modality is both more difficult and more rarely called for in everyday speech communication.

The above evidence indicates that speakers are equipped with high-level expertise in integrating audio and visual speech signals. In theory, access to fully-specified visual self-feedback (as opposed to an abstraction or novel representation like those discussed above) could be immediately useful for real-time control: connecting visual information to linguistic representations would not be any more difficult for self-image than for the image of any other speaker. In fact, it was recently shown that speakers are actually *better* at integrating visual information from recordings of themselves than of other talkers. Specifically, Tye-Murray and colleagues found that talkers were more accurate at lipreading (Tye-Murray et al., 2013) and gained more audio-visual benefit in noise (Tye-Murray et al., 2014) when perceiving from videos of themselves than of other speakers. This self-speech visual benefit was present regardless of each speaker’s baseline visual speech clarity or general perceptual abilities (Tye-Murray et al., 2014). The possibility that participants’ memories from production were the cause of the benefit, rather than the object of perception being their own faces/voices, was considered and ultimately rejected by the authors (Tye-Murray et al., 2013). It appears, therefore, that it is not only possible for speakers to connect full visual speech information to their self-produced articulation, but also that connection may be easier or more accurate than in typical perception of others’ audiovisual speech.

Together, these two bodies of literature suggest that visual information can play a role in real-time speech motor control despite its lack of availability in the typical speaking context. Novel sources of information can be learned and integrated, as the successes in speech pathology (e.g., Bernhardt et al., 2005), singing (Hoppe et al., 2006), and non-native language acquisition (e.g., Bliss et al., 2018) attest. Naturally-occurring visual feedback in the form of a mirror has also been beneficial in a wide range of therapeutic contexts, and may be useful for speakers in more typical speaking contexts, without therapeutic guidance needed, if skills from audio-visual perception of others can be applied directly to self-produced signals.

## 2 Experiment 1

In Experiment 1, we investigated two hypotheses related to feedback control in the somatosensory and visual modalities discussed above. First, we tested the possibility that topically-applied benzocaine in the supra-laryngeal vocal tract could cause sufficient perturbation of somatosensory feedback perception to impact speech intelligibility. Second, we investigated whether speakers facing degradation of feedback in other domains could use naturally-occurring visual self-feedback to recover from possible intelligibility deficits.

**FIGURE 2:**
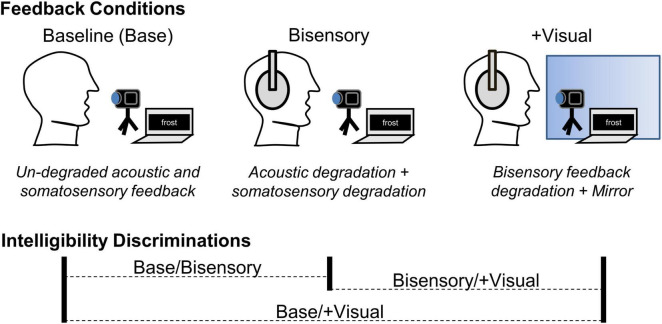
Summary of feedback manipulation conditions and listener intelligibility discriminations in Expt. 1. At baseline, acoustic and somatosensory feedback were un-degraded and visual feedback was not available. In the bisensory degradation condition (Bisensory), acoustic and somatosensory feedback were degraded. In the + Visual condition, the bisensory feedback degradations were maintained, but visual feedback was made available by the addition of a mirror. When listeners were asked to discriminate between the relative intelligibility of two tokens, all three possible condition combinations were used, comparing baseline to Bisensory and baseline to + Visual, as well as Bisensory to + Visual, in randomized relative order.

We explored both research questions in a three-condition, repeated-measures study where we manipulated the sensory information available to speakers, beginning with an unperturbed baseline (see Figure 2). Following baseline recordings, we applied benzocaine gel to each speaker’s lips and tongue blade to degrade somatosensory input in the second session. Immediately afterward, while the benzocaine effects were still present, a third session was conducted in which speakers were given real-time visual self-feedback in the form of a mirror. Recordings of speech produced in each condition were then used in an intelligibility discrimination task with a non-overlapping set of naïve participants.

Our investigation of benzocaine as a somatosensory feedback perturbation was motivated by its potential to imitate the anesthetic methods used in Larson et al. (2008), Kleber et al. (2013), and De Letter et al. (2020), but in easily accessible vocal tract structures and using a widely available, common topical oral anesthetic at a concentration that can be obtained without a medical prescription. Benzocaine is frequently used in clinical and dental medicine settings, is sold over-the-counter in the United States in concentrations of up to 20%, and produces anesthetic effects through absorption in the mucus membranes of the oral (or pharyngeal/laryngeal) cavity and interference with the sodium ion channels that facilitate potentiation in somatosensory nerve cells (Alqareer et al., 2006; Lee, 2016; Singh et al., 2024). There have been questions concerning its effectiveness for pain reduction in medical applications (Nusstein and Beck, 2003), and it is not as potent as lidocaine in limiting sensation (Lee, 2016), meaning some somatosensation may still occur under benzocaine application.

The degradation – but not elimination – of somatosensory speech feedback provides an interesting parallel to acoustic feedback degradation, where acoustic signals can be eliminated, masked, or altered, but bone-conducted sensation remains relatively constant (as shown in Figure 1 above). Previous anesthetic speech feedback perturbations eliminated somatosensory sensation (Larson et al., 2008; De Letter et al., 2020), but such complete absence of feedback may influence control parameters differently than a degradation or muting of sensory availability. Given the likely-incomplete interruption to somatosensation provided by over-the-counter topical benzocaine, and the robustness of acoustically-based control in speech, we believed the degree of degradation would likely be insufficient in isolation to cause systemic changes to production. If isolated application of benzocaine had resulted in no observable effects on speech intelligibility, for example, the null result could be attributed either to insufficient somatosensory interference or to the efficacy of acoustic feedback in maintaining control under adverse conditions. For this study, therefore, we chose to conduct an initial test where the efficacy of acoustically-based control was limited by a simultaneous degradation in the auditory domain, so that speakers would not have the option of relying on normal, fully-specified acoustics to compensate for loss of information in the somatosensory domain.

Previous studies have investigated the effects of real-time degradation of acoustic spectral information as an auditory feedback perturbation. Casserly (2015), e.g., used noise-based vocoding to simulate the signal processing of a cochlear implant (e.g., Schvartz and Chatterjee, 2012; Shannon et al., 1995) in speakers’ real-time auditory feedback. This transformation reduced the overall frequency range of auditory feedback as well as eliminating fine-grained spectral detail (see Method below). When speakers’ feedback was degraded in this way, vowel acoustic contrast diminished (Casserly, 2015) and listeners judged the speech to be less intelligible overall (Casserly et al., 2018). Given the magnitude and novelty of the vocoding degradation in these studies, it is possible that speakers experiencing this manipulation interpreted the acoustic signal as “other-produced,” rather than an altered form of their self-produced speech acoustics. Although such an attribution might seem problematic in a feedback manipulation, there is reason to believe that the acoustic signal would influence speakers’ response regardless of its source, as long as it was identified as speech (cf. Bradshaw et al., 2024; Lametti et al., 2014).

For Experiment 1, we chose to replicate both the auditory feedback degradation and the intelligibility discrimination judgment task from Casserly et al. (2018). Because the effects of the specific degradation were previously documented, this design allowed us to compare the present combined effects of acoustic degradation and benzocaine application with the single-sense degradation result in the literature. If benzocaine was sufficient to cause speech-relevant degradation of somatosensory feedback, we hypothesized that speakers’ intelligibility would decline relative to their own unperturbed baseline and to the intelligibility levels seen in auditory-only feedback degradation.

In addition to allowing for straightforward comparison with auditory-only degradation from Casserly et al. (2018), the intelligibility discrimination task was selected to assess speech intelligibility for several reasons. First, it is a wholistic task, asking listeners to assess pairs of tokens and choose one of two as “easier to understand” without isolating any particular component of the utterances or making assumptions about which acoustic features should be measured in order to capture intelligibility. Second, two-alternative forced-choice tasks are considered highly sensitive for perceptual signal detection (Lau et al., 2004) and do not require the addition of background noise, as would be required to avoid “ceiling” recognition performance for our real-word stimuli. Finally, listeners’ subjective judgments of speech intelligibility have been shown to correlate strongly with intelligibility as measured by listeners’ recognition accuracy (Hazan and Markham, 2004). If differences in intelligibility judgments were found using this method, more detailed analysis of the acoustic changes occurring under benzocaine application would be warranted, along with direct testing of recognition accuracy and listener errors or confusions under adverse conditions.

The third condition in Experiment 1 was designed to test our prediction that speakers would use naturalistic visual feedback to regain intelligibility when other senses were degraded. Speakers can learn to use abstract visual feedback under atypical circumstances (Bernhardt et al., 2005; Bliss et al., 2018) and perceive speech information from their own visual image quite accurately (Tye-Murray et al., 2014), but the conditions of typical language use do not necessarily create a need for additional sensory feedback. It is unclear, therefore, what visual self-feedback would do for speech motor control under normal conditions. When speakers’ other feedback is degraded, however, a need for alternative sources of control information has been created, and visual feedback has the potential to meet that need. The second condition of the present study provided such a degradation of auditory (at least) and somatosensory feedback to create pressure for speakers to improve their speech motor control accuracy. The third condition, where naturalistic feedback was provided by a mirror, gave speakers the opportunity to use the visual feedback to regain intelligibility. If speakers could effectively use the mirror as a naturalistic, untrained source of visual feedback, as we hypothesized, then we expected to see improvements in the third condition relative to the second, as the mirror is introduced.

In summary, Experiment 1 used three speaking conditions to test two relatively novel methods of manipulating non-auditory speech feedback. Benzocaine was tested as a means of selectively perturbing oral somatosensory feedback in speakers with simultaneous acoustic feedback degradation, and the availability of naturalistic visual feedback was subsequently tested as a means for recovering control accuracy when other senses were degraded. In both cases, control effects were assessed via perceptual intelligibility judgments from naïve listeners. These tests were not meant to be definitive regarding somatosensory or visual feedback integration in speech, or to identify the particular acoustic or articulatory effects of the feedback perturbations, but to suggest fruitful paths for future speech feedback research outside the auditory modality.

### 2.1 Method

The design and manipulations of Expt. 1 are summarized in Figure 2. Using a repeated-measures design, participants produced a set of meaningful, isolated English words across three sensory feedback conditions: (1) baseline, with un-degraded acoustic and somatosensory feedback and no available visual feedback; (2) bisensory degradation, with simultaneous acoustic and somatosensory feedback degradations and no available visual feedback; and, (3) degradation + visual availability, with acoustic/somatosensory degradation but also with real-time visual feedback provided by a mirror in front of participants. Having the same participants complete all three feedback perturbation conditions allowed us to control for potential individual differences in the relative weighting of somatosensory feedback and the degree of experience with visual self-feedback during speech production (e.g., exposure to self-view in video conferencing platforms) that might occur across independent participant groups. Recordings of speech produced across all three conditions were used in a perceptual intelligibility judgment study, where naïve listeners heard two tokens of a word from the same speaker and were asked to choose which was “easier to understand.” Selection rates different from chance were taken as evidence of a difference in intelligibility across conditions. Although no acoustic analyses of the speech are reported here, the repeated-measures design allows for such analyses to potentially be conducted in the future.

#### 2.1.1 Feedback perturbation participants

Fifteen speakers of English (male *n* = 8, female *n* = 7; mean age 19.5 years) were recruited from Trinity College in the United States. Participants all passed an audiometric screening (ANSI calibrated Ambco 650A) with ≤30 dB hearing level between 500 and 8000 Hz on the day of the study and did not report a history of speech or hearing difficulties. Thirteen participants were monolingual native speakers of North American English; two were bilingual (Hebrew and Hausa with English). Procedures were approved by the Trinity Institutional Review Board, and participants were compensated for their time with $10 or academic credit.

#### 2.1.2 Stimulus materials

Participants were shown orthographic prompts for a set of 139 English words, repeated across conditions. Items were selected from Hoosier Mental Lexicon database (Nusbaum et al., 1984), and were all rated as highly familiar to US undergraduate students. The set was balanced in token frequency, with 45 highly-frequent items (≥319 tokens/million), 45 common items (97–150 tokens/million), and 49 uncommon items (6–7 tokens/million; Nusbaum et al., 1984). Within these criteria, items were selected to maximize the use of labial articulatory gestures, containing the segments [m, p, b, f, v, ∫, w, r, i] (note: in the International Phonetic Alphabet (IPA), the American English rhotic is represented as [ɹ], but we use the more common representation [r] in the present text).

These segments, which incorporate labial closure, labiodental near-closure, lip rounding, and lip spreading gestures, generate somatosensory information from the lips and are also highly salient in visual speech information (Fisher, 1968). Target segments were likely loci of changes brought on both by application of benzocaine to the lips and by making visual self-feedback available to speakers. Items were chosen to incorporate these segments at relatively high rates and with balance across lexical locations. Specifically, in each token frequency category there were six items with target segments at word onset, 11 (13 for uncommon) elsewhere in the word, and 12 items not containing target labial gestures, for a total of 139 words.

#### 2.1.3 Acoustic degradation

Acoustic feedback was perturbed by means of real-time spectral degradation imitating the signal processing in a cochlear implant. Specifically, we used a portable real-time vocoder (PRTV; Casserly, 2015; Casserly et al., 2011; Smalt et al., 2013) with an 8-channel noise vocoding algorithm. The PRTV hardware consisted of circumaural noise occluders (Elvex SuperSonic) with a lapel microphone (Williams MIC090) fixed to the headband of the occluders above the participant’s right ear, and noise-occluding insert earphones (Etymotic HF5) worn beneath. The participant’s voice was detected by the lapel microphone, sent to a solid-state processor (iPod A1367) that performed the vocoding transformation using custom software, and transmitted back to the insert earphones with less than 10 ms delay (Casserly, 2015).

The signal transformation (Kaiser and Svirsky, 1999) applied a series of eight bandwidth filters between the frequencies of 252 and 7000 Hz. Signal amplitude envelopes within each band were calculated, then used to shape band-filtered white noise of the same frequency ranges. The resulting amplitude-matched noise bands were then summed and played back to participants. As a result of this transformation of the airborne acoustic signal, frequency information within each of the eight semi-logarithmically spaced bands was lost, along with any signal above or below the 252–7000 Hz analysis range. Speakers still received unperturbed feedback via bone conduction, however, and this source of acoustic information was not disrupted. The result of the (airborne) acoustic transformation, therefore, was a degradation of overall quality and distinctiveness, rather than a masking or an introduction of explicit error.

#### 2.1.4 Somatosensory degradation

Somatosensory feedback was perturbed via topical application of 20% benzocaine suspension gel (Iodent, United Exchange Corp.). Approximately 0.2 mL of benzocaine gel was applied to the fingertip of each participant, with instruction to apply it to their lips and the tip/blade area of the tongue. Application was observed by a researcher. Following application, participants were asked to maintain an open oral posture for 1 min, ensuring that the gel would be absorbed prior to contact with salivary fluids. The onset of action for a 20% benzocaine preparation is approximately 30 s, with full penetration in 2–3 min (Singh et al., 2024). In our protocol, the 1 min. open posture period was followed by a verbal confirmation of consent to proceed with the participant, then entry to the recording booth, the onset of acoustic degradation, and the departure of the research assistant (see 2.1.5 below); all together, benzocaine efficacy should have been maximal around the beginning of the bisensory degradation condition (2 min post-application). The remainder of the study’s two degraded-feedback conditions lasted between 11 and 13 min (5–6 min for each elicitation period and 1 min for configuration changes to visual feedback; see 2.1.5 below), for a total benzocaine exposure period of 13–15 min. Benzocaine’s duration of action in oral applications has been reported as 5–15 min (Lee, 2016), although consistent anesthetic effects have also been reported for periods exceeding 120 min with concentrations one-quarter of the current strength (Weinstein, 1977). It is therefore reasonable to assume our speakers experienced maximal anesthetic effects for the first sensory degradation condition (within 5 min of action onset) and likely persistence for the remaining condition (degradation + visual feedback), although numbing may have begun to weaken for some participants.

#### 2.1.5 Procedure

Participants’ speech was recorded in a double-walled sound attenuating booth (Whisper Room) using a stand-mounted microphone (Audio-Technica AT4041) placed at a distance of 0.96 m on a small table. A video camera (Pansonic HC-WX970) was placed adjacent to the stand mic, along with a 13 in. laptop computer 0.642 m from the participant. Orthographic prompts were displayed on the laptop screen in white text on a black background. Each prompt was visible for 2.0 sec, with 500 ms between prompts, and appeared in a randomized order that was held constant across participants and conditions. Once begun, each round of stimulus elicitation lasted 5–6 min.

In the first condition (baseline), participants followed the above procedure exactly, with no modification of their sensory feedback. After completing the baseline recordings, participants were fitted with the PRTV (with the signal transformation switched off) and performed the benzocaine application and 1 min. post-application wait period. With somatosensory degradation in place, they re-entered the recording booth and the acoustic transformation was switched on. Orthographic prompts for condition 2 (bisensory degradation) began 10 s after the researcher departed the space and the door to the booth was closed; in total, approximately 2 min passed between initial application of the benzocaine and the onset of the production task.

Following completion of the bisensory degradation condition, the researcher entered the booth and introduced a large mirror to the recording environment. Specifically, a 36 in. x 36 in square mirror with 1 in. beveled edges was present on the table top behind the laptop screen and completely covered by a black, matte cloth during the first two recording periods. With the cloth covering removed, the mirror covered most of the wall space behind the table and afforded a view of the participant’s face and upper torso, along with the back wall of the booth. Portions of the mirror were occluded by the laptop screen, video camera, and microphone, but each participant’s face was clearly visible (protocol included adjustment of the configuration to achieve visibility of the face where necessary, but such adjustment was not needed). Once the mirror covering was removed and visibility checked, the researcher left the booth and orthographic prompts began for the final recording condition (degradation + visual).

Participants were fully informed of all study methods at the beginning of the session, and instructed to speak “as naturally as possible,” with no particular mention of how the various sensory perturbations might impact the task, and no instruction to maximize clarity.

In all three conditions, speech was recorded digitally (16-bit, 4.6 kHz, Marantz PMD661) over the course of a production session and segmented by hand into individual word tokens. No other editing of the recorded speech (e.g., for amplitude across speakers or tokens) was applied. These tokens were then used as stimuli in a perceptual intelligibility study to determine whether intelligibility changed as a result of the feedback manipulations.

#### 2.1.6 Intelligibility discrimination judgments

A non-overlapping set of young adults (*n* = 30; 8 male, 21 female, 1 unreported gender; mean age 18.8 years) were recruited from Trinity College to participate in a perceptual intelligibility discrimination study. They received either monetary compensation or academic credit. All perceptual participants passed a hearing screening with ≤30 dB hearing level and reported no history of hearing difficulties. Not all participants were native speakers of English, but all were highly fluent and used English almost exclusively in their everyday lives.

Participants completed a two-alternative forced-choice task in which they heard two recorded tokens of an item with its orthographic text displayed on a computer screen, and were asked to indicate which token was “easier to understand.” Tokens within a trial were always from the same speaker and from different conditions. Order of presentation of the two tokens was randomized for each trial. A string of trials, therefore, would have been something like: a baseline/visual availability comparison from Speaker 8, followed by a bisensory degradation/visual availability comparison from Speaker 2, etc. with the selection of tokens and the order of presentation fully randomized. Participant responses, ‘f’ key presses indicating the first token, ‘j’ key presses for the second, were recorded along with reaction time on each trial. Experiment presentation and data collection were done with a combination of E-Prime (Psychology Software Tools) and PsychoPy (RRID:SCR_006571; Peirce, 2007) software packages.

It was not possible for listeners to respond to the full set of tokens collected from speakers. A subset of tokens were therefore selected for use as stimuli in the perceptual judgment study. The stimulus set consisted of nine unique word items, with three tokens (representing the three sensory conditions) each, for each of the 15 speakers. For each speaker, the items were balanced across frequency of occurrence and target segment locations – including items without labial target segments anywhere in the word. For the resulting 135 items, listeners completed one trial with each possible condition comparison (see Figure 2). The order of these 405 trials was randomized for each participant.

#### 2.1.7 Data analysis

Participants were forced to select one of two tokens as “easier to understand” on every trial. If intelligibility were equivalent across conditions, therefore, selection rates would approximate 50%; if tokens of one condition or another were selected as “easier to understand” more frequently, selection rates would differ from 50% in either direction. There were three comparisons being tested in this experiment, as summarized in Table 1.

**TABLE 1:** Summary of perceptual intelligibility comparisons for Experiment 1, along with the scoring used to code each participant response to the two-alternative forced choice task.

Comparison	Condition A		Condition B	
	Description	Comparison-specific scoring	Description	Comparison-specific scoring
Base vs. Bisensory	Baseline (no degradation)	1	Bisensory feedback degradation	0
Bisensory vs. + Visual	Bisensory feedback degradation	0	Bisensory degradation + visual feedback	1
Base vs. + Visual	Baseline (no degradation)	1	Bisensory degradation + visual feedback	0

Presentation order was randomized across trials; conditions are designated as “A” or “B” only in order to parallel the labeling convention for each comparison type.

In order to test whether rates were statistically different from 50% for each comparison, we coded each participant response according to the schedule shown in Table 1, where selection of the condition with the highest-quality feedback received a one and selection of the lower-quality feedback condition received a zero. Mean responses to each condition type were then calculated for each participant, reflecting the rate at which that listener selected the higher-quality feedback condition in each comparison.

One-mean *t*-tests were conducted for each comparison type (Base/Bisensory, Bisensory/+Visual, and Base/+Visual), testing the pool of participant averages against a reference of 50%. Comparisons with rates significantly different than 50% were taken to show a significant difference in intelligibility in favor of whichever condition was selected at the higher rate.

Our primary research questions in this study centered on the effects of benzocaine as a somatosensory degradation and the effect of visual feedback availability when other senses are degraded. To test these questions as directly as possible, two additional analyses were planned. First, the relative effect of the addition of visual feedback was tested by comparing the discrimination rates in the Base/Bisensory and Base/+Visual conditions in a paired-samples *t*-test; if the difference from baseline was different across these two comparison types, the difference could be attributed to the addition of the visual feedback in the latter condition.

In order to examine the effect of benzocaine on intelligibility, a comparison was also planned between listener responses to the Base/Bisensory comparison and a condition reported in Casserly et al. (2018), where participants discriminated the intelligibility of baseline speech vs. auditory-only feedback degradation speech. The same recording conditions were used in Casserly et al. (2018), with the same auditory degradation and subsequent perceptual study design, and participants were drawn from the same population. The only difference was that the feedback-degraded speech in Casserly et al. (2018) was produced with auditory degradation only and the bisensory degradation speech in the present study was produced with an additional benzocaine application. Differences in the intelligibility effects would be reflected in greater deviation from 50% (chance) selection rates – differences in the degree of distinction between unperturbed baseline speech and the perturbation condition. In order to compare these two sets of data, therefore, we conducted an independent-samples *t*-test using participant mean selection rates in Base vs. Bisensory as one pool and mean selection rates from the Base vs. Audio-only degradation comparison (*n* = 39) reported in Casserly et al. (2018) as the other.

Un-adjusted *p*-values and 95% confidence intervals are both reported below, along with descriptive statistics. All statistical analyses were conducted in JASP (Version 0.9.1.0). The equality of variance assumption for the independent-samples *t*-test was met (Levene’s test, *F* = 1.28, *p* = 0.262).

### 2.2 Results

**FIGURE 3:**
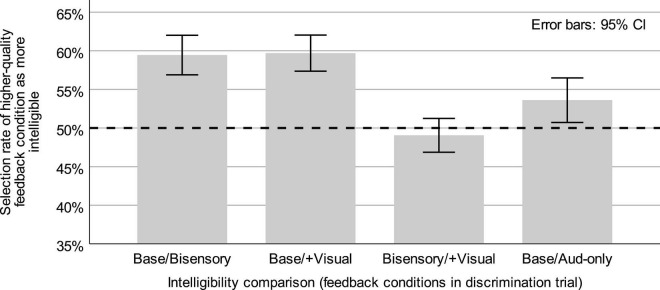
Means and 95% confidence intervals (error bars) for intelligibility discrimination responses in Expt. 1 (Base/Bisensory, Base/+Visual, Bisensory/+Visual) and for responses from a parallel task reported in Casserly et al. (2018) comparing un-degraded baseline and auditory-only degradation speech intelligibility (Base/Aud-only). Dashed line represents chance discrimination.

Means and 95% confidence intervals for the selection rates of the higher-quality feedback condition (see Table 1) for each comparison type in Expt. 1 and the auditory-only degradation comparison in Casserly et al. (2018) are shown in Figure 3.

In intelligibility discrimination trials containing only degraded-feedback speech, with tokens from the bisensory degradation and degradation + visual feedback conditions, the higher-quality feedback condition (degradation + visual feedback) was selected as “easier to understand” at a mean rate of 49.06% (*s* = 5.87%), with a 95% CI of [46.86, 51.25] for the mean. This selection rate did not differ significantly from 50% (*t*(29) = −0.88, *p* = 0.386).

Trials asking listeners to discriminate between either of the degraded-feedback conditions and the unperturbed baseline speech were also analyzed in order to determine whether there were consistent contrasts in perceived intelligibility. In trials containing speech from the baseline and bisensory degradation conditions (Base/Bisensory comparison), listeners selected tokens from the unperturbed baseline as “easier to understand” at a mean rate of 59.45% (*s* = 6.87%). This rate was significantly above chance (*t*(29) = 7.54, *p* < 0.0001), with a 95% CI of [56.89, 62.02] for the mean. In trials comparing speech from the baseline and degradation + visual feedback condition (Base/+ Visual comparison), the baseline was also frequently selected as “easier to understand,” with a mean baseline selection rate of 59.71% (*s* = 6.27%), with a 95% CI of [57.36, 62.05] for the mean. This rate was also significantly above chance (*t*(29) = 0.848, *p* < 0.0001). Following these single-group analyses, the mean higher-feedback selection rates (see Table 1) were compared across the Base/Bisensory and Base/+Visual conditions to determine the effect of additional visual feedback on judgments of intelligibility. Descriptive statistics for each group were reported above, with mean selection rates of 59.45 and 59.71% for the baseline over the bisensory and bisensory + visual degradation conditions, respectively, with a mean difference of −0.26 and a 95% CI of [−2.03, 1.53] for the mean difference. This difference across the comparison types, a difference corresponding to the availability of real-time visual feedback, was not significant (*t*(29) = −0.29, *p* = 0.774).

Additionally, the selection rate data from the Base/Bisensory condition was compared to parallel data from Casserly et al. (2018) where listeners responded to a comparison of unperturbed baseline speech and speech produced with auditory feedback degradation but no simultaneous benzocaine application (Base/Aud-only comparison). The mean selection rate of baseline speech as “easier to understand” in the Base/Aud-only comparison was 55.45% (*s* = 8.82%), with a 95% CI of [52.88, 58.31] for the mean, and which differed significantly from chance (*t*(38) = 3.86, *p* = 0.0004). A comparison of those selection rates from the current Base/Bisensory selection rates, where both auditory and somatosensory feedback were degraded, showed a significantly larger preference for the baseline (a larger intelligibility difference) in the Base/Bisensory condition (*t*(67) = 2.05, *p* = 0.044), with a difference in means of 4.0%, a 95% CI of [0.12, 7.90] for the difference in means. The Cohen’s *d* effect size for this result was 0.498.

#### 2.2.1 Bayesian statistics comparison

Of the results reported above, two do not allow for rejection of the null hypothesis under conventional inferential analysis, and one other case produced results quite proximal to the rejection decision criterion at *p* = 0.05. In such cases, it can be informative to explore analysis from the Bayesian perspective, which compares the likelihood of various models instead of focusing on a null hypothesis rejection criterion (Kruschke, 2015). Briefly, Bayesian statistics take a prior distribution of probability across the models, consider the fit of the observed data with both models, and shift the prior distribution according to that fit, generating a “posterior” distribution of probability for each model. The posterior probabilities are reflected in a set of Bayes factors, which indicate the relative posterior probability of one model over the other reciprocally: one, called BF_0_ here, represents the probability of the null hypothesis model over the probability of the alternative; and the other (BF_alt_) represents the opposite, the probability of the alternative hypothesis model over the probability of the null.

In model-comparison Bayesian analysis, the accuracy of the posterior probabilities (and therefore the Bayes factors) depends on the nature of the prior distributions for each model. That is, the specific characteristics of these models and their output predictions matter for the accuracy of the Bayesian output. An overly-broad prior, in particular, can skew the posterior probabilities either in favor of the null hypothesis or away from it, depending on the nature of the data (Kruschke, 2015; Vanpaemel, 2010). It is therefore recommended that prior distributions be as specific as possible, given the actual hypotheses of the study. One result of this recommendation is that, if there is a directional hypothesis for a study, researchers should test that hypothesis directly in Bayesian statistics, rather than using two-tailed, non-directional analyses as in null-hypothesis significance testing. In significance testing, one-tailed tests are avoided because they increase the rate of Type I error. In Bayesian statistics, the specificity of the prior makes the analysis a more stringent comparison of the two model types (but the prior must be accompanied by a genuine, theory-driven explanation for its directionality).

We have applied Bayesian statistical analysis to three of the questions described in the null hypothesis significance testing results above: the Bisensory/+Visual comparison to chance discrimination, the comparison of discrimination rates in the Base/Bisensory and Base/+Visual conditions, and the comparison of the Base/Bisensory condition and the Base/Aud-only degradation condition in Casserly et al. (2018). In each case, we specified a directional prior for the alternative hypothesis, as described below. The rationale for these priors was the same in all cases: when more feedback was available (i.e., from an un-degraded sensory source), we predicted that intelligibility would be higher.

In the case of the Bisensory/+Visual discrimination data, we compared the probabilities of a null hypothesis model centered at 50% selection rate (chance) to an alternative hypothesis model specifying a selection rate of the + Visual condition that was greater than 50% due to higher feedback availability (all priors were specified as Cauchy distributions with width 0.707; robustness checks showed no crossovers at any width for any analysis). As reported above, the mean selection rate of + Visual speech as more intelligible was 49.1%. One-mean Bayesian analysis returned a BF_0_ = 8.93 (BF_alt_ = 0.11) in favor of the null hypothesis model centered at 50%. Specifically, according to the BF_0_ the null is 8.93 times more likely to have generated these data than the alternative hypothesis.

For the non-significant comparison between the Base/Bisensory and Base/+Visual responses above, we again specified a directional prior in the Bayesian statistical equivalent. In this case, the degree of feedback availability was poorer in the bisensory degradation condition than in the degradation + visual feedback condition, so the contrast in intelligibility with un-degraded baseline speech should have been greater in the Base/Bisensory comparison than the Base/+Visual comparison. Both prior hypotheses specified a mean degree of difference between groups; the null model was centered at zero, the alternative specified that the Base/Bisensory group selection rates should be greater than those of the Base/+Visual (a positive, non-zero as the group difference). As described above, the mean selection rates for each condition were 59.5% (Base/Bisensory) and 59.7% (Base/+Visual). Paired-comparison Bayesian analysis returned a BF_0_ = 6.33 (BF_alt_ = 0.16). The null hypothesis was therefore 6.33 times more likely than the alternative, given these data.

Finally, we examined the critical comparison between Base/Bisensory discrimination data in the present study with Base/Aud-only degradation data from Casserly et al. (2018). Because the bisensory degradation condition was identical to the auditory-only degradation condition except for the application of benzocaine, participants in the current study should have had poorer feedback availability, and therefore a greater change in intelligibility relative to baseline, than participants in the auditory-only condition. Our alternative hypothesis model accordingly specified that the Base/Bisensory comparison should be greater (further from 50%) than the Base/Aud-only condition. Our null hypothesis model was once again centered at zero (no difference in intelligibility). Independent-samples Bayesian analysis returned BF_alt_ = 2.83 (BF_0_ = 0.35), favoring the alternative hypothesis as 2.83 times more likely than the null, given these data.

### 2.3 Discussion

This experiment sought to test the effects of two non-auditory feedback manipulations on speech intelligibility: application of benzocaine as a somatosensory degradation, and availability of real-time visual self-feedback as a visual feedback augmentation.

#### 2.3.1 Benzocaine as feedback degradation

Using a combination of conventional and Bayesian statistics, Experiment 1 showed support for the capacity of topically-applied, commercially-available preparations of benzocaine to provide a speech-relevant degradation of somatosensory information. Not only did the bisensory degradation condition, with simultaneous auditory and somatosensory manipulations, produce speech that was judged to be significantly less intelligible than “normal” baseline speech, but also the difference from baseline was shown to be greater than what has been observed with auditory degradation alone. The contrast between these two conditions (bisensory degradation and auditory-only degradation) was not numerically large (4.0% difference in mean selection rates of the baseline as more intelligible), but all aspects of the analysis (significance testing, confidence intervals, effect sizes, and Bayesian likelihoods, as well as a preliminary acoustic analysis reported in Casserly et al., 2016), support that it is a contrast worth consideration. It therefore seems reasonable to conclude that the application of benzocaine caused a small but genuine decrease in participants’ speech intelligibility – beyond what would be expected with auditory degradation alone.

The efficacy of commercially-available benzocaine preparation as a non-invasive manipulation of sensory feedback opens the door for new, broadly accessible investigations into somatosensory feedback perturbation, without requiring special medical personnel or supervision. Although we have seen that complete or near-complete elimination of tactile feedback through prescription-strength lidocaine can disrupt speech motor control sufficiently to cause categorical errors in consonants and changes in articulation rate (De Letter et al., 2020), the articulatory and acoustic-phonetic consequences of a less complete degradation are still unknown, along with the effect of differential degrees of anesthesia across the articulators, and the applicability of somatosensory feedback perturbation results to a diverse pool of participants. The ability to conduct studies outside of a medical setting makes these research avenues accessible to a greater breadth of researchers and may therefore facilitate the data collection needed to address them.

As with any methodological innovation, however, there are questions to answer as well. In particular, there is variation in the efficacy of all topical oral anesthetics, depending on the pH of the application environment, the duration of the application, and the specific preparation method, among other factors (Meechan, 2000). Variation in this domain would impact the reliability of feedback manipulation results, the statistical power obtainable across speakers, and the ability to interpret individual differences in speech behavior. For example, it has been hypothesized that speakers may weigh feedback from the auditory and somatosensory modalities differently (Lametti et al., 2012). Somatosensory degradation experiments could provide converging evidence for this dimension of variation, but only if the degree of degradation were well-controlled across speakers. Otherwise, differences in apparent feedback weighting could instead be caused by differences in the degree of sensory manipulation experienced by participants. Other factors, such as the diffusion or specificity of the area of physiological effect and variation in the duration of effects, would also ideally need to be described before the usefulness of the method could be fully leveraged. With those caveats in mind, however, the method could have substantial impact on the study of speech-relevant somatosensory information.

#### 2.3.2 Real-time visual speech feedback

Due to the ubiquitous benefits of visual speech information for perception of others and the use of abstract visual information as a source of feedback-based control, we hypothesized that speakers would be able to use real-time visual self-feedback from a mirror as a means of improving their production accuracy when other sources of sensory information were degraded. This hypothesis was not supported; intelligibility discrimination was at chance when listeners were directly comparing tokens from the bisensory degradation and degradation + visual feedback conditions, and the degree of difference from baseline in each condition was highly similar. Bayesian statistics confirmed that these data provided stronger support for null hypotheses reflecting no effect of visual feedback (or rather, no difference between the second and third speaking conditions) than our theoretically-motivated alternatives by factors of 6–9 times. It appears, therefore, that speakers in our study did not use the available visual feedback to improve their speech intelligibility.

There are at least three possible explanations for the lack of visual feedback benefit seen in this study: first, that speakers generally cannot use visual information of this type in speech motor control without specific instruction or therapeutic guidance; second, that speakers shifted to use feedforward control strategies, obviating the need to integrate the relatively novel feedback at all; or third, that speakers attempted but were unable to use the visual information in this *particular* context.

Regarding the first possibility, results from self-lipreading and self-targeted speech in noise perception suggest that recognition of speech targets is facilitated for self-generated signals relative to other-generated signals (Tye-Murray et al., 2013, 2014), demonstrating not only that participants can use visual self-speech to perform speech perception, but also that self-generation of signals is a benefit to recognition rather than a barrier. Tye-Murray and colleagues take this facilitation as evidence of a “common code” underlying speech representations, one that is accessible through any sensory modality that provides information relevant to a perceived production event, regardless of its frequency of use or occurrence (Tye-Murray et al., 2013, 2014). Such an interpretation suggests that linking visual speech information to similar linguistic targets of real-time motor control should be easily within the capacity of speaker/listeners, without particular guidance. If speakers generally cannot make this perceptual connection in real-time control, such a finding would run directly counter to common code theories, as least insofar as they apply to the targets of feedback-based control. Moreover, the fact that specific instructions are typically provided as part of mirror use in a speech therapeutic setting does not necessarily mean that such instruction would play a crucial role here. Ben-David and Icht (2018), for example, did not report specific instructions to their speakers regarding use of a mirror for an oral-diadochokinesis task, yet exposure influenced speech production for both young and older adults. Further, speech feedback manipulation effects in the acoustic domain have been shown to be highly robust, regardless of explicit instruction or speaker intentions (Lane and Tranel, 1971; Munhall et al., 2009). It therefore seems unlikely that speakers in our study *could* have used the available visual feedback effectively, but did not do so due to the lack of specific instruction.

It is also possible, however, that the bimodal degradation of sensory information caused speakers to decrease their reliance on real-time feedback altogether, relying instead on established articulation motor plans in a strictly feedforward control strategy. Such down-weighting of unreliable feedback is predicted by influential speech motor control models such as DIVA (Guenther and Vladusich, 2012) and the state feedback control model (Houde and Nagarajan, 2011). It is not clear, however, why a lack of reliability in one sensory domain (or two, as in the present case) would negatively impact the influence of a fully-reliable alternative sensory stream. Moreover, when Chesters et al. (2015) investigated the effects of delayed speech feedback across the auditory and visual modalities, it was found that visual speech feedback in the form of a digital mirror influenced speakers only when the paired auditory feedback was also perturbed. That is, when auditory feedback was not delayed, a delay in visual self-feedback had no effect on speaking rate, intensity, pitch or disfluencies and errors (Chesters et al., 2015). When auditory feedback was delayed, however, a similar temporal shift in visual feedback magnified the deleterious effects, beyond what was observed with delayed auditory feedback alone (Chesters et al., 2015). The results provide an interesting parallel to the present study, in which intact visual feedback also did not result in recovery from perturbed auditory feedback, but Chesters et al. (2015) further demonstrated that visual feedback was not being ignored completely, and exerted significant influence on articulatory control under the right circumstances – the equivalent of which presumably were not present in our Experiment 1.

The final potential explanation for our results is that participants could have used the visual feedback, or possibly even attempted to do so, but the simultaneous degradation of the auditory and somatosensory streams negatively influenced participants’ ability to use the visual feedback provided. We cannot know the absolute degree of degradation achieved by our manipulations, but if they interfered sufficiently to impact intelligibility, then they may also have interfered with participants’ ability to execute speech production adjustments based on the intact visual stream. Even if the bisensory degradation only increased the variability of compensatory efforts, rather than systematically negatively impacting their execution, we might expect to see a lack of effect of visual feedback in the aggregate speech behavior. Moreover, the increased processing demands associated with two independent sources of perceptual adversity may have taxed the control system such that novel visual feedback could not be effectively integrated. Adverse listening conditions for other-targeted perception have been associated with high levels of cognitive demand or “listening effort” (Anastasios et al., 2009; Wagner et al., 2016), and such demands can cause performance on other, simultaneous tasks to decrease. Visual self-feedback is relatively novel for speakers and may therefore be demanding to process (even if the end result of such processing could be highly accurate). If visual feedback integration is demanding, then it may not have been possible for our speakers to perform integration under such difficult conditions.

The distinction between a general inability to use real-time visual self-feedback, an inhibition of all sensory feedback in response to widespread degradation in the acoustic and somatosensory domains, and an interference effect (of either articulatory control or processing demands) is theoretically important. If speakers cannot use the novel source of feedback under any conditions, then extensive modality-specific and task-specific learning must be necessary for successful feedback-based control. However, if speakers could use the visual feedback, but the articulatory processes or cognitive demands of simultaneous bisensory degradation prevented them from doing so, or caused such feedback to be ignored in favor of a feedforward control strategy, then such modality- and task-specific learning may not be required. Given the importance of contrasting these explanations for our findings in Experiment 1, we designed a second experiment to investigate the effects of real-time visual self-feedback when no other sensory manipulation was in place.

## 3 Experiment 2

This experiment largely replicated the method and procedures from Experiment 1, but with only two speaking conditions: (1) baseline, when speakers could hear and feel themselves normally but could not see themselves speaking; and (2) +mirror, when the mirror used in Expt. 1 was placed in front of participants to make naturalistic real-time visual self-feedback available. A new group of naïve listeners made intelligibility discrimination judgments on speech produced across the two conditions in order to assess the effect of the visual feedback on intelligibility.

There were three possible outcomes: improvement as a result of visual feedback, reduction in intelligibility, or no change. If speakers could successfully use the novel feedback source to augment existing speech motor control, we expected improved intelligibility in the +mirror condition. There is room to increase intelligibility in normal speech, as evidenced by speakers’ ability to produce so-called “clear speech” (Ferguson and Kewley-Port, 2007), though there is variability in speakers’ success, and also the hyper-articulated speech associated with the Lombard effect (Van Summers et al., 1988), etc. If speakers cannot use visual feedback effectively, however, even when all other task demands have been removed, then we would expect to see no change in intelligibility or even a negative impact as speakers tried and failed, e.g., to integrate the visual information in control.

### 3.1 Method

The method used in Expt. 2 was identical to that of Expt. 1 except for the following changes.

#### 3.1.1 Participants

No participants were involved in both Expt. 1 and Expt. 2 or in both the speaker and listener roles in the present study. Thirteen speakers were recruited from the Trinity College student body (6 male, 6 female, 1 unreported gender; mean age 19.2 years) and passed an audiological screening on the day of participation (≤30 dB hearing level between 500 and 8000 Hz) and did not report a history of speech or hearing difficulties. For the intelligibility judgment portion of the study, an additional 23 participants were recruited (4 male, 18 female, 1 unreported; mean age 20.2 years) and screened to verify normal hearing on the day of the study. As in Expt 1., not all participants were native speakers of English, but all were highly fluent and used English almost exclusively in their everyday lives. All participants in both portions of the study received either monetary compensation or academic credit for their time, and all aspects of the study design were reviewed and approved by the Trinity College IRB.

#### 3.1.2 Procedure

The procedure for Expt. 2 consisted of only two conditions, rather than three, and no sensory degradations were applied. The only change from the first condition to the second was the placement of a large (36′′ × 36′′) mirror in front of the speaker, in the position described for the degradation + visual feedback condition in Expt. 1 above. All other elements were the same as for Expt. 1.

#### 3.1.3 Intelligibility discrimination judgments

Because there were only two speaking conditions instead of three, listeners only needed to make one discrimination judgment for each word item. The reduction in number of comparisons (and two fewer speakers, relative to Expt. 1) meant that we could include a larger number of words from each of our talkers. To approximate the overall study length of Expt. 1 and maintain a balance across word frequencies and other dimensions of control, 32 words were selected from each speaker for use in the perceptual discrimination study. Listeners responded to the baseline/+mirror comparison for all 32 words from each speaker, for a total of 384 trials.

#### 3.1.4 Data analysis

Once again, selection rates in the intelligibility discrimination task were compared against random chance, 50%, in order to assess whether speech produced in one condition or the other was systematically preferred by listeners in terms of ease of understanding. In the case of a null result, we planned to use Bayesian statistics to differentiate between data supporting a 50% discrimination rate versus data that was inconclusively or variably consistent with other alternatives. Based on the assumption that more speech-relevant sensory information should lead to greater control accuracy, we predicted that speech in the +mirror condition would be more intelligible than speech produced at baseline. For the standard *t*-test analysis, however, a two-tailed test was conducted to reduce the likelihood of Type I error.

### 3.2 Results

**FIGURE 4:**
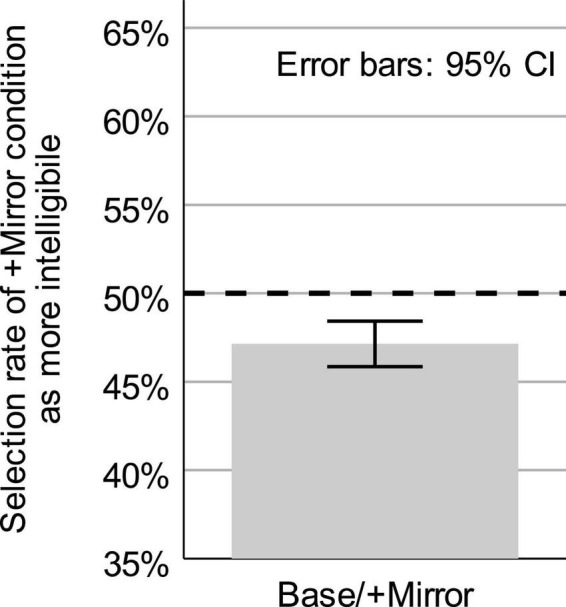
Mean and 95% confidence interval (error bars) for the rate of selecting the +Mirror condition as “easier to understand” in the Expt. 2 intelligibility discrimination task. Comparison was between baseline speech with no immediate visual feedback to speech produced in front of a mirror (+Mirror). Dashed line represents chance discrimination.

The mean discrimination selection rate and 95% confidence interval for the baseline versus +mirror comparison is shown in Figure 4. When listeners discriminated between speech produced in these two conditions, they chose the higher-feedback condition (+mirror) as “easier to understand” a mean of 47.14% of the time (*SD* = 2.97%). A one-mean *t*-test found this to be significantly different from chance performance (*t*(22) = −4.619, *p* = 0.00013), with a 95% CI of the difference from 50% of [−4.15, −1.58]. Listeners therefore found speech produced in the baseline to be significantly easier to understand than speech produced in front of a mirror.

### 3.3 Discussion

Instead of an increase in intelligibility, which would be expected if speakers were able to use naturalistic visual feedback under these more favorable conditions, we observed a significant *decrease* in intelligibility when real-time visual feedback was provided. As described above, such a negative impact of visual feedback suggests that speakers were ineffective in either their integration or processing of this novel information, but also that the visual feedback did exert some effect on speech motor control despite the robustness of other sensory modalities. Given the evidence for successful use of similar visual information in tasks other than real-time control (Tye-Murray et al., 2013, 2014), it would seem that the learning required for feedback-based motor control must be specific to that purpose, and speakers cannot capitalize on experience in other areas. However, this conclusion stands in direct opposition to the observation that perceptual training influencing acoustic-phonetic vowel category boundaries can impact the degree of speakers’ adaptation to formant perturbations (Lametti et al., 2014). Moreover, there is evidence of the reverse generalization, of perceptual learning from feedback control influencing broader, other-targeted speech perception (Shiller et al., 2009). The largest distinction between these findings and the present study, other than a focus on the auditory rather than visual domain, is that the perceptual learning in both cases was experimentally-induced and therefore relatively brief compared to the lifelong learning of visual speech characteristics. This distinction may be crucial for generalization, or the demands of using perceptual information for purposes of real-time control in the visual domain may make it too task-specific to be a good target, as opposed to source, of generalization.

It was striking, however, that participants’ speech intelligibility in this experiment was actually judged to worsen as a result of the introduction of visual feedback. Intact sources of feedback information in the auditory and somatosensory domains would ideally protect against such deleterious effects, even if speakers were attempting (ineffectively) to use visual feedback to aid in control. That is, there was nothing in Experiment 2 preventing speakers from detecting their reduction in intelligibility and correcting for it. Moreover, there was no overt motivation for speakers to change their speech production at all when the mirror was introduced; no instructions were given concerning speech clarity or effort. Participants only knew they were part of a study investigating how sensory information interacted with “how people talk,” not any of the specific hypotheses or questions involved. The cause of speakers’ sacrificed intelligibility, therefore, is not clear. It does, however, replicate a similar deleterious effect reported by Ben-David and Icht (2018) for young adult speakers completing an oral-diadochokinesis task with and without visual feedback from a mirror. In their study, speakers were asked to repeat the non-word [pataka] as quickly as possible in 5-s intervals; rates of accurate repetition were shown to increase with practice when no mirror was present, but young adults who were shown their face in a small mirror during non-initial intervals did not improve over time (Ben-David and Icht, 2018). Ben-David and Icht (2018) hypothesized that the presence of unnecessary sensory information could have “strain[ed] cognitive resources, thus impairing performance.”

There is some evidence from the social and cognitive psychology literatures that mirrors can influence behavior more broadly, e.g., priming participants’ knowledge of external evaluation of their behaviors and causing them to endorse more pro-social beliefs (Wiekens and Stapel, 2008) and avoid socially-condemned actions like cheating and littering (de Kort et al., 2008; Diener and Wallbom, 1976). There are also reports that people find their reflections aversive, experiencing a negative reaction to viewing themselves in a mirror (Rochat and Zahavi, 2011). If mirrors have these additional influences on participants, it is possible that the processing involved in these effects was itself responsible for depleting the resources available for the speech production task. That is, speech intelligibility has been shown to decrease as a result of speakers performing a simultaneous task (Harnsberger et al., 2008). If viewing themselves in a mirror was sufficient to engage other processing, such as inhibition of attention to the aversive stimulus or split attention to potential external evaluation, or other generalized distraction, speakers may have shown decreased intelligibility as a result of that “dual task,” rather than any direct influence of the feedback on motor control. This experiment cannot definitively determine whether this dual-task interference is causing the negative intelligibility judgments in response to speech produced in front of a mirror. The question is therefore open for future research.

## 4 General discussion

Across two studies, we investigated the effects of naturalistic visual feedback and topical anesthetic application as manipulations of visual and somatosensory speech feedback, respectively.

Although there were strong theoretical motivations to predict that speakers would be able to use a naturalistic source visual self-feedback to aid in real-time motor control, we saw no evidence consistent with that prediction. Instead, we observed no change in intelligibility when other senses were degraded (Expt. 1) and a negative effect when other feedback sources were intact (Expt. 2). These observations are consistent with the predictions of models of speech motor control like the DIVA (Guenther and Vladusich, 2012), which incorporate modality- and control-domain-specific learning as a critical component of control. Specifically in DIVA, the basis by which speakers relate sensory feedback and speech production relies upon experience-dependent, detailed mappings between articulation and sensory information. Under such a model, the extensive visual-speech perceptual experience of our talkers would not be helpful for use in motor control unless it were paired with similarly extensive experience relating self-produced articulatory movements to visual speech percepts; one piece (perceptual experience) without the other (sensorimotor mapping) would not be enough.

The most straightforward interpretation of our results for visual feedback, therefore, supports the importance of control-specific perceptual learning. There is a possibility that feedback integration from the relatively novel mirror source was sufficiently demanding to result in decreased intelligibility due to dual-task interference (Harnsberger et al., 2008), but direct evidence of such interference and its relationship to the kinds of changes seen in speech with other dual-task interference would be needed to support such a conclusion.

Tentatively, therefore, we conclude that the results of these studies support theories of speech motor control that specify modality-dependent learning of sensorimotor mappings as the basis for feedback use. Visual feedback provides an interesting test case for exploration of these models, given the relative lack of experience in sensorimotor speech mapping in this modality, on the one hand, and the plentiful technological opportunities for generating experience in such mappings on the other. Speakers have more opportunities than ever before to see themselves while speaking, e.g., during videoconferencing, and these technological applications may open an avenue to exploring visual sensorimotor speech learning in adults in new and theoretically-enlightening ways.

In contrast to the complex results observed for visual speech feedback, the results for our somatosensory feedback manipulation were relatively straightforward. In Experiment 1, topically-applied benzocaine was found to be sufficient to cause speech-relevant degradation of somatosensory feedback, as seen in intelligibility deficits greater than those observed with auditory degradation alone (Casserly et al., 2018). Although analysis of the specific acoustic changes leading to listeners’ judgments is outside the scope of the current paper, this method of degradation has promise for wide application in the field of speech motor control, we believe, due to its relative ease of execution, the lack of simultaneous changes to articulatory configuration along with somatosensation, and the potential for differential application of the anesthetic gel to specific articulatory structures (e.g., degrading feedback from the tongue blade but not the lips, or vice versa).

There are important questions to be answered about the effects of benzocaine on somatosensation, however, especially concerning variability in efficacy. More information on variation in the degree of impact with constant dosage, the area affected by particular areas of application, and the timecourse of degradation effects would all be beneficial in improving experimental control in investigations using this method. The results here represent a first step, therefore, pointing out that the gathering of such information may be worth the effort in terms of the potential benefits for feedback perturbation research.

## 5 Conclusion

In order to understand the process of real-time speech motor control in depth, probing the ways in which speakers use and integrate non-auditory sources of sensory feedback is crucial. In this study, we have identified an accessible, speech-relevant means of manipulating the quality of somatosensory feedback and investigated speakers’ ability to use naturalistic visual self-feedback from a mirror, finding that there are barriers to its effective use in articulatory control. Identifying the precise nature of those barriers, whether related to a lack of modality-specific learning or to external draws on cognitive resources, may further illuminate the role of experience in the development of effective speech motor control. Regardless of the cause, the visual feedback difficulties highlight the importance of conducting speech feedback research outside the auditory domain, where issues of experience, generalization, and cross-modal integration may differ across the senses or across tasks. Speech is inherently a multimodal phenomenon, and our experiments in real-time articulatory control need to continue to reflect that reality in the methods they employ.

## Data Availability

The datasets presented in this study can be found in online repositories. The names of the repository/repositories and accession number(s) can be found below: Open Science Framework (OSF), doi: 10.17605/OSF.IO/RSWZE.
